# Synthesis and evaluation of ^99m^Tc-DOTA-ARA-290 as potential SPECT tracer for targeting cardiac ischemic region

**DOI:** 10.22038/IJBMS.2021.57565.12799

**Published:** 2021-11

**Authors:** Naser Mohtavinejad, Maliheh Hajiramezanali, Mehdi Akhlaghi, Ahmad Bitarafan-Rajabi, Nazila Gholipour

**Affiliations:** 1 Faculty of Pharmacy, Baqiyatallah University of Medical Sciences, Tehran, Iran; 2 Department of Radiopharmacy, Faculty of Pharmacy, Tehran University of Medical Sciences, Tehran, Iran; 3 Research Center for Nuclear Medicine, Tehran University of Medical Sciences, Tehran, Iran; 4 Echocardiography Research Center, Rajaie Cardiovascular Medical and Research Center, Iran University of Medical Sciences, Tehran, Iran;; 5 Cardiovascular Interventional Research Center, Rajaie Cardiovascular Medical and Research Center, Iran University of Medical Sciences, Tehran, Iran; 6 Chemical Injuries Research Center, Systems Biology and Poisonings Institute, Baqiyatallah University of Medical Sciences, Tehran, Iran

**Keywords:** ARA-290, Erythropoietin (EPO), Ischemia, Molecular imaging, Technetium-99m

## Abstract

**Objective(s):**

Myocardial infarction caused by ischemia of heart tissue is the main reason for death worldwide; therefore, early detection can reduce mortality and treatment costs. Erythropoietin (EPO) has protection effects on ischemic tissue due to nonhematopoietic peptide (pHBSP; ARA-290) which is derived from the B-subunit of EPO.

**Materials and Methods:**

We designed and synthesized a modified DOTA-(Lys-Dabcyl^6^, Phe^7^)-ARA-290 using Fmoc solid-phase peptide synthesis strategies. To improve serum stability, Fmoc-Lys-(Dabcyl)-OH as lipophilic amino acid was synthesized along with Fmoc-Phe-OH which then were substituted with Arg^6^ and Ala^7^, respectively; they were then investigated for the ability to detect ischemic cardiac imaging. DOTA-(Lys-Dabcyl^6^,Phe^7^)-ARA-290 was labeled with technetium 99m, and its radiochemical purity (RCP), stability in the presence of human serum and, specific bind to hypoxic H9c2 cells were evaluated. *In vivo* studies for biodistribution and SPECT scintigraphy were checked in a normal and cardiac ischemia rat model.

**Results:**

Radiolabeling purity was obtained more than 96% by ITLC, and in vitro stability of the radiopeptide up to 6 hr was 85%. The binding of ^99m^Tc-ARA-290 to hypoxic cells was remarkably higher than normoxic cells (3 times higher than normoxic cells at 1 hr). Biodistribution and SPECT imaging on the cardiac ischemic model showed that radiopeptide considerably accumulated in the ischemic region (cardiac ischemic-to-lung rate = 3.65 ID/g % at 0.5 hr).

**Conclusion:**

The results of studies,* in vitro* and *in vivo*, indicated that 99mTc-DOTA-(Lys-Dabcyl6,Phe7)-ARA-290 could be an appropriate candidate for early diagnosis of cardiac ischemia.

## Introduction

Myocardial infarction, the most prominent cardiovascular disease outcome (CVD), is the main reason for morbidity and death in the world (1). Immediate and precise diagnosis of cardiac hypoxia and ischemia is very critical and crucial for appropriate treatment and prevention of myocardial damage (2). Evaluation of cardiac ischemic biomarkers in blood plasma, including Creatine Kinase-MB, cardiac troponin, and lactate dehydrogenase (LDH) is valuable for diagnosis; however, their levels increase in blood after a delay of time (3). Hence, early detection will decrease direct and indirect medical expenses and mortality. Erythropoietin (EPO) is a 31 kDa glycoprotein hormone secreted from the liver into the embryonic and puberty period kidneys, respectively for promoting the differentiation and growth of red blood cells by the bone marrow and is made of 4 α-helices subunits (A–D). Subunit helix B is hydrophilic and appears to be associated with tissue protection by binding the EPOR-BcR complex receptors (4). 11 amino acids out of 85 in B helix with sequences Pyr-Glu-Gln-Leu-Glu-Arg-Ala-Leu-Asn-Ser-Ser-OH act as a pharmacophore. This peptide was introduced to the Helix-B Surface Peptide (ARA-290) with a half-life of roughly 2 min in rats and rabbits (5, 6). During the hypoxia and ischemia incidence, the hematopoietic cytokine erythropoietin receptor (EPOR-BcR) was joined together by signaling and up-regulated in the cell surface (7). The hypoxic cells will be induced to regeneration, frustrate apoptosis and restrain inflammation by activating principle signaling of JAK2, STAT-5, and PI3K/Akt pathway (8). Due to conducted studies in different hypoxia models, such as critical limb ischemia (CLI) through immunohistochemistry and Western blot tests, EPO-BcR receptor is up-regulated in hypoxia conditions. Besides, the ARA-290 peptide reduces CLI symptoms (9).

Willingness to use single-photon emission computed tomography (SPECT) and positron emission tomography (PET) allowed the noninvasive quantity and powerful molecular imaging of regional physiological processes such as myocardial blood flow (SPECT) in human beings which is widely used for the diagnosis and monitoring of disease. (10). In radiopharmaceuticals, *in vivo* stability is a crucial factor to design new peptides (11,12). Many studies were done to enhance the half-life of ARA-290 with the addition of a thioester bond and PEGlation (13). 

In the present study, we attempted to increase *in vivo* stability of ARA-290 peptide by replacing lipophilic Lys-(Dabcyl)-OH derivatives and Phe-OH amino acid in place of Arg^6^ and Ala^7^, by the Fmoc solid-phase peptide synthesis process, respectively. The peptide that increased lipophilicity compared with the parent peptide can improve its half-life by changing interaction profiles human serum albumin (HSA), distribution volume, cells penetration, and phospholipid affinity (14). Peptides were directly labeled with [^123^I], [^125^I], and [^18^F] or indirectly (chelator assisted) with [^99m^Tc], [^111^In], and [^64^Cu] radionuclides as a radiotracer in SPECT or PET imaging (15). Technetium-99m was used for the half-life balance matching with peptide clearance, simply accessibility and cost-effectiveness, excellent imaging quality, and favorable dosimetry (16). For the peptide labeling with Technetium-99m various chelators such as bifunctional chelating agents (DTPA, NOTA, DOTA, and PCTA), PnAO (N_4_) diamine dithiol (N_2_S_2_), and HYNC were used. In this research tetraazacyclododecane-1,4,7,10-tetraacetic acid (DOTA**)** was used as a BFCA (17). 

In this study, we synthesized Fmoc-Lys(Dabcyl)-OH as an amino acid derivative and inserted it into the DOTA-ARA-290 peptide sequence during synthesis. The main purpose of this research was to evaluate *in vitro* and *in vivo* features of ^99m^Tc-DOTA-(Lys-Dabcyl^6^, Phe^7^)-ARA-290 for early diagnosis of the ischemic cardiac region and to determine the level of exertion in normal and ischemic rats.

## Materials and Methods

***Reagent***

Fmoc-protected amino acids and 2-Chlorotrityl chloride resin (100-200 mesh, loading: 0.6 mmol/g) were bought from Sigma Aldrich (MO, U.S.A.). Fmoc-Lys(Dabcyl)-OH as a modified amino acid was synthesized according to Shen *et al*.’s procedure (18). All necessary materials required for the synthesis of Fmoc-Lys(Dabcyl)-OH including 4-amino benzoic acid, dimethylaniline, L-lysine hydrochloride, Fmoc-OSu, and Ethylenediaminetetraacetic acid (EDTA), were bought from Sigma Aldrich (MO, U.S.A.). The DOTA-NHS-ester as a chelator was purchased from Macrocyclic (TX, U.S.A). Other chemical agents, including DIEA, HOSu, TBTU, and DCC were taken from Sigma-Aldrich (MO, U.S.A.) and applied without further purification. The infrared spectrum was recorded on a Perkin Elmer Spectrum BX-II spectrometer. ^1^H-NMR spectra were recorded in dimethyl sulfoxide-d^6^ (DMSO-d6) by a Bruker 500 MHz instrument (Billerica, Germany). Analytical reverse-phase HPLC was used for purification of the synthesized peptides. (Knauer, Germany) 1000-PU with a 50 ml/min pump head equipped with a UV detector 2500 and wavelength range of 190–740 nm. CC 250/4.6 Nucleosil C18 column and 120/ 20 Nucleosil C18 column were used for analytical and preparative HPLC, respectively. The mobile phase consisted of 0.1 % trifluoroacetic acid in 100 % acetonitrile (Solvent A) and 0.1% trifluoroacetic acid in 100% water (Solvent B), and the gradient flow rate of 1.0 ml/min at an A:B ratio of 30:70 at 0.01 min, 55:45 at 25 min, 100:0 at 25.1 min, and was stopped at 30 min. VaCo 5-11 used the Freeze-drying feature of the purified product (Zirbus technology, Germany). Mass spectra (*m/z*) were determined on positive electrospray ionization (ESI^+^). Heart myocardium H9c2 cells (Nanjing, China) were cultured in Dulbecco’s modified Eagle’s medium (DMEM) at 37 °C. Sodium pertechnetate (Na ^99m^TcO⁻₄) freshly milked from a commercial ^99^Mo/^99m^Tc generator (Radioisotope Division, NSTRI, Tehran, Iran). Radiochemical purity was calculated by thin-layer chromatography paper (SG-TLC); (silica gel 60; Merk). Measuring gamma was done on an ORTEC Model 4001 M c-system well counter. Animal trial and *in vivo* experiments were according to in the Animal Care and use Committee of the Baqiyatallah University of Medical Sciences guidelines. (# IR. Bums.97000030). 

***Chemical synthesis of Fmoc-Lys(Dabcyl)-OH as amine acid derivative***

*Synthesis of 4-((4-(dimethylamino) phenyl) azo) benzoic Acid (Dabcyl; I)*

For this purpose, 4-aminobenzoic acid was chosen to prepare diazonium salt (19). 4-aminobenzoic acid (6.8 g, 50.0 mmol) and sodium hydroxide (2 g, 50.0 mmol) were blended in water (25 ml) with stirring. Aqueous sodium nitrite solution (3.5 g, 50.0 mmol in 25 ml water) was added to this solution dropwise at 0–4 °C. After blending for 10 min, a solution of hydrochloric acid (25 ml of 8 wt %, 50.0 mmol) was added. The mixture was put in an ice-salt bath and quickly mixed for about 25 min. To the orange paste as diazonium salt, we added N,N-dimethylaniline (6.05 g, 50.0 mmol) dissolved in NaOH solution (4 g at 10 wt %, 50.0 mmol). The resultant compound was tightly mixed for 1 hr, and the saturated solution of NaOAc was added to the product to adjust the pH. The obtained mixture was filtrated by filter paper and washed with a large amount of water, and dried at room temperature to reduce product impurities. The crude product **Ι** (6.8 g, 69% yield) was obtained as an orange paste and used for the next step of the synthesis.^ 1^H NMR, 500 MHz, DMSO-d6: δ; 3.08 (s, 6H, N (Me)_ 2_), 6.84 (d, *J*= 8.55 Hz, 2H, H_3’, 5’_), 7.81-7.84 (m, 4H, H_3, 5_, H_2’, 6’_), 8.07 (d, *J*= 8.15 Hz, 2H, H_2, 6_) ppm. Elem. Anal. Elem. Calcd for C_15_H_15_N_3_O_2_: C, 66.90; H, 5.61; N, 15.60; Found: C, 66.59; H, 5.48; N, 11.78.

*Synthesis Dabcyl-OSu (IIa)*

To a cooled THF (30 mL, 0–2 °C), crude **I** (1.2 g, 4.65 mmol) and DCC (1.10 g, 5.3 mmol) as coupling agent were added, and N-hydroxysuccinimide (HOSu) (0.56 g, 4.9 mmol) was added to this solution. After one hour of mixing at freezing temperature, the compound was stirred overnight at ambient temperature. The product was filtrated to eliminate dicyclohexylurea impurities and then dried and crystallized with acetone-diethyl ether under a vacuum situation. The product was Dabcle-N-hydroxysuccinimide ester (Dabcyl-OSu) **IIa** (1.3 g, 84%). 

***Synthesis N*** ^ƹ^ ***-Dabcyl-lysine (IIb)***

These groups were chelated with copper cation to hinder α-NH_2_ and α-COOH lysine from the reaction with **IIa**. L-lysine hydrochloride (0.6 g, 2.7 mmol) was dissolved in water (10 ml); afterward, CuCO_3_.Cu(OH)_2_.H_2_O (0.45 g, 1.80 mmol) was added to this solution. The mixture was blended at a boiling temperature under reflux conditions. After two hours of heating, the undissolved cupric carbonate was extracted from the warm mixture by filtration; then, it was washed with boiling water (10 ml). After cooling, NaHCO_3_ (0.22 g, 2.48 mmol) and then the crude (**IIa)** (0.9 g, 2.46 mmol) dissolved in DMF (25 ml) was added dropwise to the cold cupric complex at 0–5 °C under strong stirring over a period of about 2 hr. After stirring for 24 hr, the compound was filtered, and before being dried, the precipitate was washed by water, acetone, and ether sequentially. Finally, to remove Cu, the powdered complex was dissolved in plenty of saturated Ethylenediaminetetraacetic acid (EDTA) solution and stirred overnight at ambient temperature. The precipitate (**IIb)** was extracted by filtration, washed with water, and then air-dried (0.74 g, yield 82%). 

*Synthesis of N* ^ƹ^ *-dabcyl-N* ^α^ *-(9-fluoreny1methoxy)-carbonyllysine (Fmoc-Lys (Dabcyl)-OH; III)*

At this stage, Fmoc was bound as a protector of α-NH_2_ to N^ƹ^-dabcyl-Lysine. To a cold solution of CH_3_CN / DMF / H_2 _O (6:2: 1) crude **IIb** (1.2 g, 2.9 mmol) was added and mixed at 0-2 °C. The pH of solution was set at 8-8.3 by addition of NaHCO₃ (0.24 g, 3 mmol), then Fmoc-OSu (1.3g, 3.94 mmol) was slowly added within an hour. The reaction mixture was taken out two times with ether (30 ml). Then, by the addition of 5 ml of 6 M hydrochloric acid, the aqueous layer was acidified to pH=3. The raw product was chromatographed (chloroform /methanol = 50: 1) to obtain 1.68 g of pure 1 (yield 48%). The mixture was checked by TLC- silica gel plate (chloroform /MeOH = 5:1, *R*_f_ = 0.3). ^1^H NMR, 500 MHz, DMSO-d6: δ; 1-48-1.53 (m, 2H, CH_2_), 1.71 (d, *J*= 6.85 Hz, 2H, CH_2_), 1.88-1.94 (m, 2H, CH_2_-_diastrotopic_), 3.23 (s, 6H, N(Me)_2_), 3.51 (m, 2H, CH_2_), 3.59 (s, 1H, CH), 4-13-4.16 (m, 1H, CH), 4.29-4.40 (m, 2H, CH_2_-_diastrotopic_), 6.75 (d, *J*= 8.55 Hz, 2H, H_3’,5’_), 7.34-7.39 (m, 2H, CH_fmoc_), 7.41 (t, *J*= 7.50 Hz, 1H, CH_fmoc_), 7.47 (t, *J*= 7.50 Hz, 1H, CH_fmoc_), 7.55-7.56 (m, 2H, CH_fmoc_), 7.72-7.74 (m, 4H, H_3,5_, H_2’,6’_), 8.38 (d, *J*= 8.15 Hz, 2H, H_2,6_), 9.41 (s, 1H, NH) ppm. Anal. Elem. calcd for C_36_H_37_N_5_O_5_: C, 69.77; H, 6.02; N, 11.30. Found: C: 69.91, H: 6.30, N: 11.48.

***Solid-phase peptide synthesis***

The Fmoc-protected peptide was produced on 2-Colourtritely chloride resin (1.6 mmol/g) (20). In brief, the resin (1g, 1.6 mmol) was inflated in a synthesis tube by DCM (2×1), DMF (10 ml) then the binding of amino acids to each other was reacted in the presence of 3 mol excess of Fmoc-amino acid, 3 mol excess of N,N,N′,N′-Tetramethyl-O-(benzotriazole-1-yl) uranium tetrafluoroborate (TBTU) as a coupler agent and 8 mol excess of diisopropylethylamine (DIPEA) in dimethylformamide (DMF). For closing the uncoupling site, the resin was capped by DCM:MeOH:DIPEA (20:2.4:1.2). The accuracy of the amino acid coupling reaction was evaluated at the end of each step by the Kaiser test. Moreover, piperidine 25% was applied to remove the residual Fmoc. After attaching the last amino acid and before peptide cleaving from the resin, 1 mmol of DOTA-NHS-ester and 3 mol excess of TBTU in DMF (10 ml) were directly added to the reaction vessel. The peptide-chelator was isolated from the resin by using 1% TFA v/v dichloromethane (DCM). Furthermore, side-chain protective groups were excluded by a 2-h treatment cocktail consisting of trifluoroacetic acid (TFA), triisopropylsilane (TIPES), methanol, and water (90:.025:0.05: 0.025). The organic solvent residue in the product was removed under vacuum with conventional rotary then cooled diethyl ether was added. The precipitated DOTA-(Lys-Dabcyl^6^,Phe^7^)-ARA-290 was suspended in acetonitrile/TFA (99.9:0.1) and purified by RP-HPLC. The purified product was identified by ESI-MS spectroscopy.

^99m^ ***Tc-radiolabeled of DOTA-(Lys-Dabcyl*** ^6^ ***,Phe*** ^7^ ***)-ARA-290***

To assess the high radiochemical purity, various parameters were optimized, such as (i) amount of peptide-chelator complex, (ii) volume of SnCl_2_ as a reducing agent, (iii) fitting of stock in various pH, and (iv) reaction temperature (21,22). Therefore, we tested the values 5–50 μg of DOTA-(Lys-Dabcyl^6^,Phe^7^)-ARA-290 as a ligand, 10–50 μg SnCl_2_ (1 mg/ml SnCl_2_, 2H_2_O in 0.1M HCl) and 925 MBq of ^99m^TcO⁻₄ in 0.5ml saline. Also, to assess the impact of soluble pH in producing a high labeling purity, it surveyed in pH 4–9. A temperature of between 25 and 60 °C for 15 min was incubated. RCP % was determined through a well counter.

*Radiochemical study*

The radiochemical purity of ^99m^Tc-DOTA-(Lys-Dabcyl^6^,Phe^7^)-ARA-290 was measured by thin-layer chromatography (ITLC-SG) strips (23). In this way, after making 25 μl droplets of the radio-peptide in the origin of the TLC stripe, it was placed on two different mobile phases: (1) methanol: ammonium acetate 1:1 to ascertain for ^99m^TcO_2_ (R_f_ = 0.0); (2) methyl ethyl ketone (MEK) for unlabeled ^99m^TcO^-^_4_ (R_f_ = 1.0). The TLC strip was divided into 10 pieces (1 cm each) then the nuclear radiation of each segment was evaluated by a gamma counter. RCP was calculated by the following formula:

RCP = 100 - (free ^99m^TcO^-^_4_ + ^99m^TcO_2_)

*Stability studies*

As an *in vitro* stability evaluation, radiolabeled peptides (specific activity= 18.5 MBq/nmol) were added to sodium chloride 0.9 % isotonic solution (24). After incubation at ambient temperature, samples were taken out at 0.06, 0.5, 1, 2, 4, and 6 hr. The radiochemical purity of the mixture was investigated by a TLC stripe. To survey biostability, 100 μl of the labeled compound was added to fresh human serum (1 ml) and incubated (37 °C, 5% CO_2_) at different time points (4, 30, 60, 120, 240, and 360 min). 100 μl aliquots of serum samples were then incubated with 100 μl of ethanol. Samples were centrifuged at 5000 rpm for 5 min at 37 °C and 20 µl supernatant was removed to assess the degradation of ^99m^Tc labeled peptide by TLC. 

*Partition coefficient (Po/W)*

For evaluating the partition coefficient, 0.5 ml of n-octanol was added to a solution of 20 MBq ^99m^Tc-DOTA-(Lys-Dabcyl^6^,Phe^7^)-ARA-290 in 0.5 ml sodium chloride (0.9%). The test micro-tube that contained the two-phase system was left to be stirred. After 5 min mixing, phase isolation was performed by centrifugation at 5000 rpm for 5 min (25). Three samples (100 μl) and their radioactivity were calculated in a gamma counter from each layer. After getting average radioactivity, values from each of the phases were used to calculate the partition coefficients (Po/w) by dividing the radioactivity of the octanol phase to the aqueous phase.

*In vitro hypoxia test*

H9c2 myocardial cells were separated into three groups. The normoxic control group (negative EPOR-BcR receptor) was cultured in a normoxic (20% O_2_) culture medium. Hypoxic group (positive EPOR-BcR receptor) was cultured in a different time interval (1-4 hr) hypoxic culture condition (5% CO_2 _and 2% O_2_) and blocking test (specific binding assay) in the hypoxic condition.   Hanks’ balanced saline mixture (1.3 mM CaCl_2_, 5 mM KCl, 0.5 mM MgCl_2_, 0.4 mM MgSO_4_, 0.3 mM KH_2_PO_4_, 4 mM NaHCO_3_, 69 mM NaCl, and 0.3 mM Na_2_HPO_4_) without serum or glucose (26), as well as H9c2 myocardial cells were inserted into the hypoxia incubator chamber (MIC-101) that coupled to a hypoxic tank containing 5 % CO_2_, 2% O_2_, and 94% N_2_ gas (27). The cells for triplicate were placed inside confocal plates (Costar) at a density of 1 × 10^5^ per well, then treated with almost 250 pmol of ^99m^Tc-DOTA-(Lys-Dabcyl^6^,Phe^7^)-ARA-290 subject to 0.025 MBq at 37 °C. Incubation was disrupted with 1 ml of chilled PBS. Afterward, the cells were separated by 0.03 trypsin to measure the cell-bound level of ^99m^Tc-DOTA-(Lys-Dabcyl^6^,Phe^7^)-ARA-290 by a scintillator. EPOR-BcR receptor blocking was done by using 50-fold extra of the unlabeled peptide in 30 min before the injection of the labeled peptide. 

Rat models of ischemia injury

To study ischemia-induced myocardial injuries, male Wistar rats weighing 200~250 gr were sorted into three groups of four (n=4): (1) normal group, (2) Ischemic group, and (3) sham group. The rats were anesthetized by intraperitoneal (IP) administration of 3 % pentobarbital sodium (60 mg/kg).  Positive pressure mechanical ventilation was made by a Shinano respirator (SN-480–4) and an open-heart operation was done at the level of the left 4th–5th ribs. The rats received intramuscular injections of penicillin sodium (0.8 mg/g) to prevent infection. Myocardial ischemia was induced by a slipknot (4–0 silk suture) around the LAD about 2–3 mm near its origin. After 30-min ischemia, the slipknot was loosened to restore normal circulation for reperfusion (28). A pale color verified prosperous local myocardial ischemia in the ischemic area and cardiac biomarkers consisting of cardiac Creatine Kinase (CK), Troponin-I (cTnI), and CK-MB were checked in all groups. In the same group, heart surgery was performed without LAD artery occlusion.

*Biodistribution studies*

^99m^Tc-DOTA-(Lys-Dabcyl^6^,Phe^7^)-ARA-290 solution, including 20 MBq activity was intravenously injected into three groups (n=3) of male Wistar rats through their tail veins (29). The biodistribution of radiopeptide among tissues was measured by sacrifice of rats for each elected interval (30, 60, 180, and 360 min) post-injection. Various tissues, including heart, bone, kidneys, muscle, salivary glands, spleen, and intestines, were removed immediately and rinsed with saline solution to clean their residual blood. Moreover, blood samples for monitoring radioactivity were taken from the heart by a heparin-impregnated syringe after sacrificing. The percentage of injected activity per gram (%ID/g) for different organs was measured by dividing the activity of each organ per decayed total injected activity and mass of each organ. Moreover, in ischemic rats, 100 μg unlabeled peptide was co-injected with radiolabeled peptide to analyze the *in vivo* blocking test.

*Scintigraphy studies*

30 and 90 min post-injection of ^99m^Tc-DOTA-(Lys-Dabcyl^6^,Phe^7^)-ARA-290 (200 MBq) into ischemic and normal rats, planar images were captured by a dual-head SPECT system (E.cam, Siemens, Germany) that was arranged with low energy high-resolution collimators, a 128*128 matrix size, and a 20% energy window set at 140 keV (29).

Statistical analysis

Data from three replications were presented as mean ± SD. Using Student’s t-test, treatment outcomes and that of the control group test were analyzed. The standard deviation and means of the biodistribution were calculated by Microsoft Excel software (2018). Results were considered statistically significant at *P*<0.05. 

## Results

***Synthesis of Fmoc-Lys (Dabcyl)-OH as amine acid derivative***

**Scheme 1:**
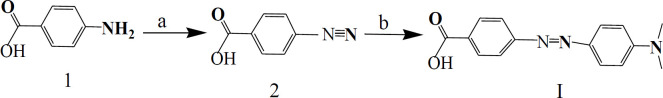
Synthesis of 4-(4-hidroxyphenylazo)-benzoic acid (**I**). Reagent and condition: a) NaOH, NaNO_2,_ HCl , 25 min , 0-4 °C; b) Dimethylaniline, NaOH, 2 °C, 1 hr, 69%

**Scheme 2:**
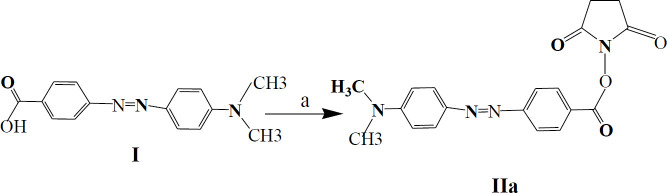
Synthesis of Dabcyl-OSu (**IIa**). Reagent and condition: a) THF, DCC, 1 hr, 0-2 °C; HOSu, 25 °C, 24 hr, 84%

**Scheme 3:**
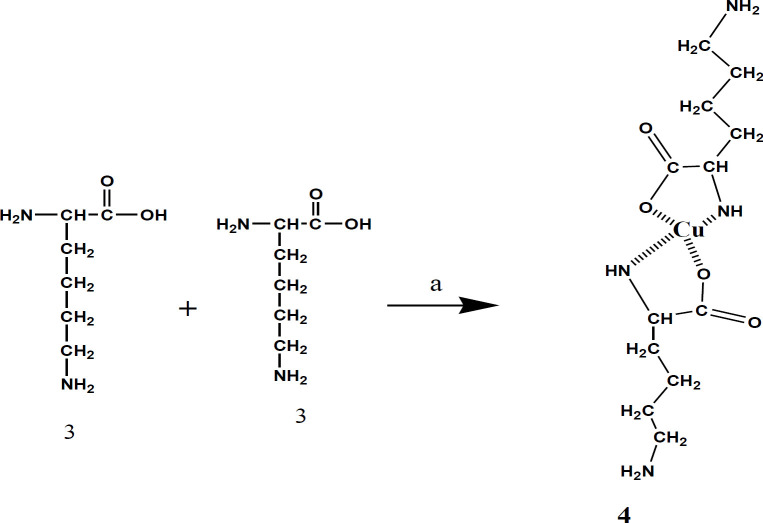
Synthesis of Lys-Cu-Lys. Reagent and condition: a) CuCO_3_.Cu (OH)2.H_2_O, 1 hr, 100 °C; H2O, NaHCO, 25 °C

**Scheme 4:**
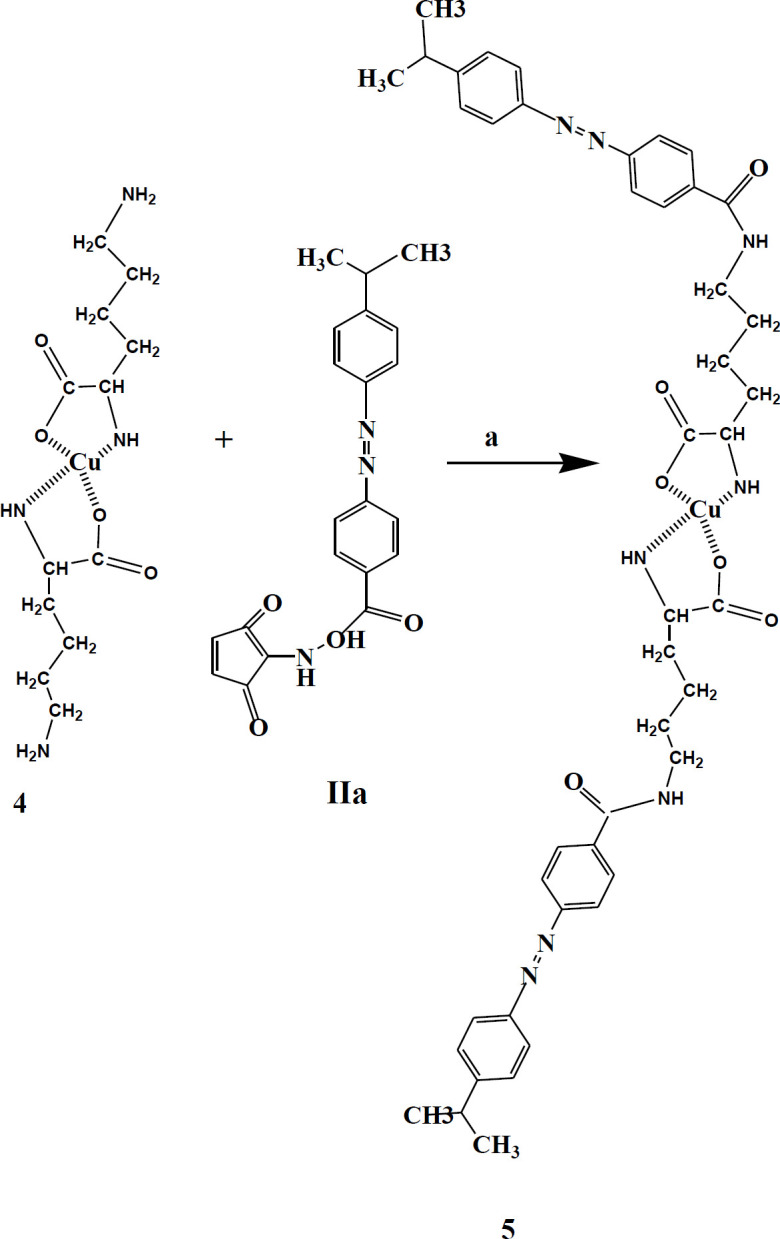
Synthesis of Dabcyl-Lys-Cu-Lys-Dabcyl. Reagent and condition: a) CuCO_3_.Cu (OH)2.H_2_O, 1 hr, 100 °C; H_2_O, NaHCO, 25 °C

**Scheme 5:**
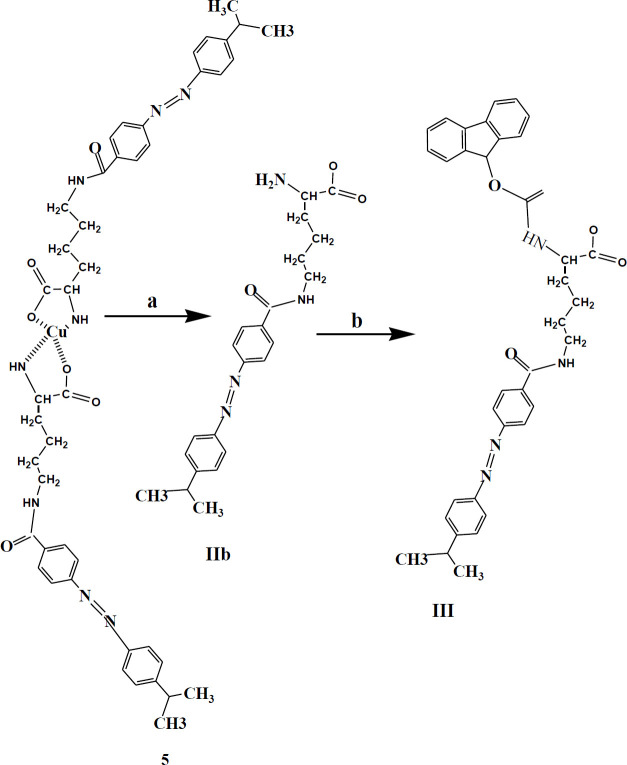
Synthesis of Fmoc-Lys (Dabcyl)-OH (**III**). Reagent and condition: a) EDTA (1×2), 24 hr, 25 °C. b) CH3CN/DMF/H_2_O (6:2: 1), 0-2 °C; Fmoc-SOu, 1 hr, 0-2 °C; HCl, Ether, 25 °C

Dabcyl (4-((4-(dimethylamino) phenyl) azo) benzoic Acid; **I**) was synthesized in a yield of 69% (Scheme 1). To increase the reactivity of the carboxyl group of Dabcyl, HOSu was added as a strong leaving group, and activated product was obtained with a high yield of 84% (Scheme 2). A detailed description of the Lys-Cu-Lys (**4**) synthesis is shown in Scheme 3 that would allow the α-NH_2_ and α-COOH of lysine hydrochloride chelation. The N^ƹ^-Dabcyl-lysine (**IIb**) was obtained with 82% purity by reacting Dabcyl-OSu (**IIa**) to the Lys-Cu-Lys mixture in an ice bath (Schemes 4 and 5).

In the final step to the usability of this amino acid derivative in the SPPS pathway, Fmoc-OSu was added to α-NH_2_ of N^ƹ^-dabcyl-Lys (**IIb**) (Scheme 5), from which Fmoc-Lys(Dabcyl)-OH (**III**) was produced in a yield of 48%. 

***Solid-phase peptide synthesis***

**Figure 1.**
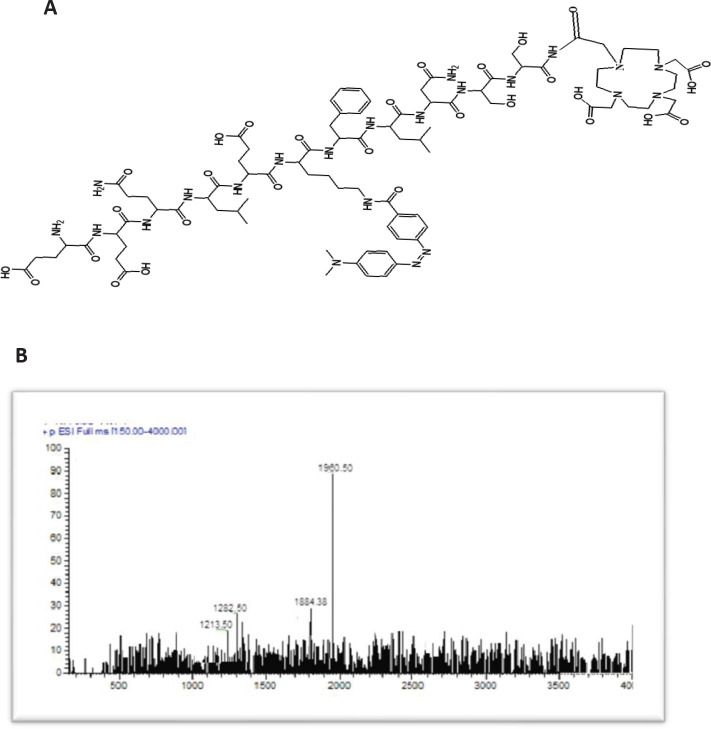
(A) The skeletal structure of DOTA-(Lys-Dabcyl^6^,Phe^7^)-ARA-290 and (B), Electrospray ionization-mass spectroscopy of DOTA-(Lys-Dabcyl^6^,Phe^7^)-ARA-290

Chelating DOTA-NHS-ester was covalently attached to modified ARA-290 that was synthesized on CTC resin applying Fmoc SPPS. The structural and molecular masses of the synthesized peptide were analyzed by HPLC–ESI-MS. The obtained purity was >97%, as verified by HPLC (Table 1). The calculated mass for DOTA-(Lys-Dabcyl^6^,Phe^7^)-ARA-290 is 1959.33 m/z, and ESI-Mass spectrometry analysis confirmed a [M+H]^ +^ molecular ion of 1960.25 m/z (Figures 1A, B).

***Radiolabeling***

**Figure 2:**
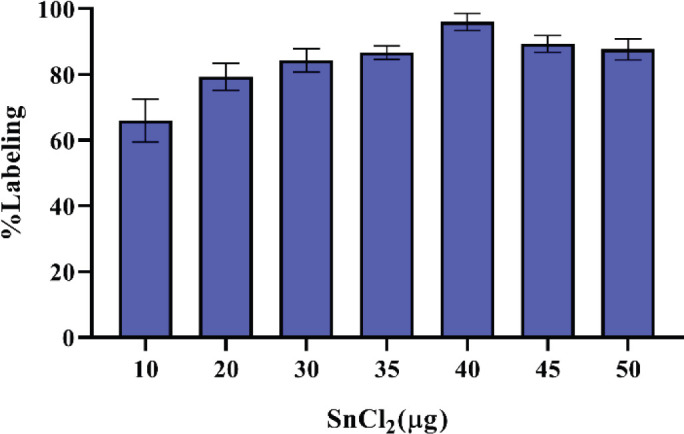
Effect of SnCl_2_ on labeling purity of^ 99m^Tc-DOTA-(Lys-Dabcyl^6^,Phe^7^)-ARA-290 (mean ± SD, n=3)

**Figure 3:**
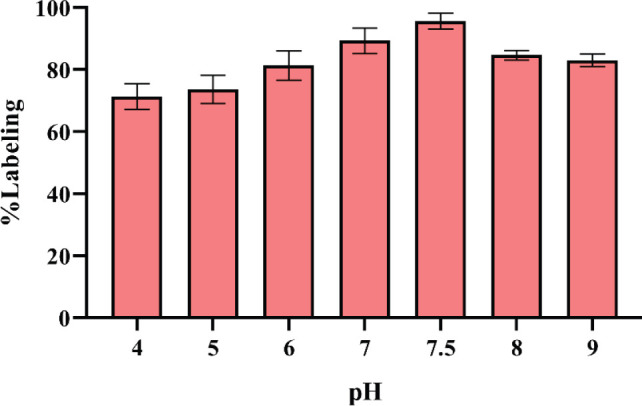
Effect of reaction pH on ^99m^Tc-DOTA-(Lys-Dabcyl^6^,Phe^7^)-ARA-290 (mean ± SD, n=3)

**Figure 4:**
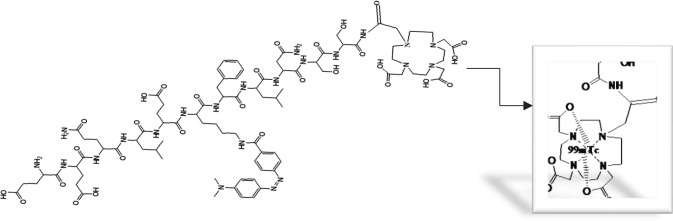
Possible complex formed between technetium-99m and DOTA

^99m^TcO^-^_4_ for labeling DOTA-(Lys-Dabcyl^6^,Phe^7^)-ARA-290 was freshly eluted from the ^99^MO/^99m^Tc generator. As shown in Figure 2, the amount of SnCl_2_ affects the labeling purity of radiopeptide. Also, the inference was that by increasing the pH around 6.5–7.5, in parallel, radiochemical purity improves (Figure 3). The amount of peptide-chelator that is used in labeling is important. As shown in Table 2, a significant decline in radiochemical purity by reducing the mass of peptide to 10 μg was seen (82.04% vs 96%). Increasing the amount of ligand up to more than 40 µg not only did not significantly increase the radiochemical purity but also reduced the specific activity. In general, the highest radiochemical purity (>%96) was obtained for 20 µg DOTA-(Lys-Dabcyl^6^,Phe^7^)-ARA-290, 40 µg SnCl_2 _(1 mg/ml SnCl_2_, 2H_2_O in 0.1M HCl), 925 MBq activity in 1 ml normal saline (pH=7.5), and incubation time of 15 min at room temperature. The possible complex that can be formed between technetium-99m and DOTA is shown in Figure 4. 

***Assessment of partition coefficient and stability***

**Figure 5:**
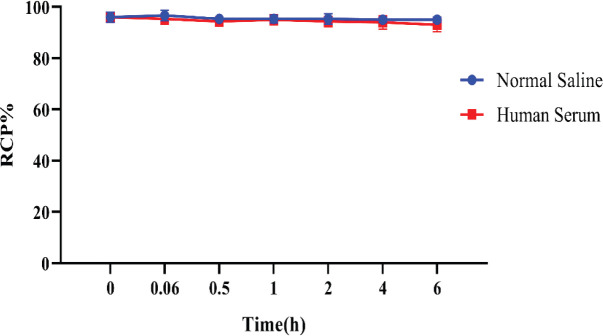
Normal saline and human serum stability of ^99m^Tc-DOTA-(Lys-Dabcyl^6^,Phe^7^)-ARA-290(mean±SD, n=3)

The saline stability of radiopeptide was 95% up to 6 hr, whereas the human serum data revealed respectively 94% and 93% in 4-6 hr after incubation (Figure 5). The results confirmed our radiolabeled peptide had suitable stability in saline and human serum for 6 hr**. **Likewise, the substance log P (lipophilicity) data was −0.66 ± 0.28%.

In vitro testing of hypoxia

**Figure 6:**
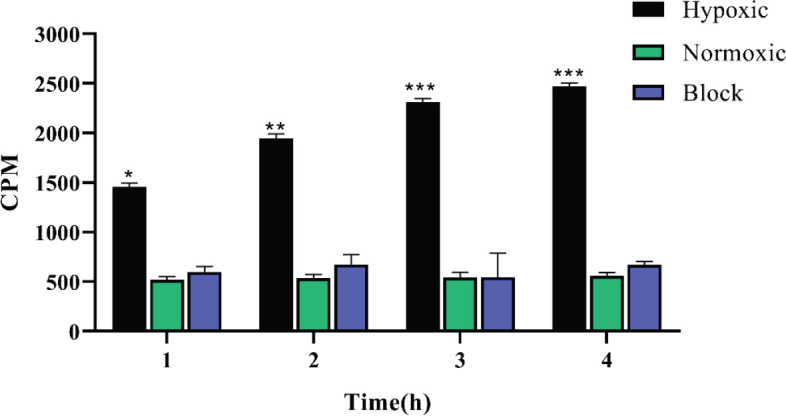
Hypoxic test of ^99m^Tc-DOTA-(Lys-Dabcyl^6^,Phe^7^)-ARA-29 for different times (1, 2, 3, and 4 hr)

To verify the up-regulation of EPO-BcR receptor in a hypoxic state and binding of synthesized radiopeptide to the receptor, Myocardium H9c2 cell lines in triplicate were incubated in hypoxia (2% O_2_) and normoxic (20% O_2_) condition at various time intervals (1, 2, 3, and 4 hr). The results presented statistically significant variations in the *in vitro* ability of^ 99m^Tc-DOTA-(Lys-Dabcyl^6^,Phe^7^)-ARA-290 in binding to hypoxic cells (Figure 6). The binding affinity of^ 99m^Tc-peptide to the H9c2 cells that were incubated under normoxic situations was very low. In contrast, radiopeptide binding affinity to the EPO-BcR receptor was increased under hypoxic situations over time (almost 5 folds at 4 hr vs 3 folds at 1 hr). In a blocking test that used 50 mol excess of the unlabeled peptide, 30 min before adding the radiopeptide to the hypoxic cells, the same result was obtained for the normoxic cells. Thus, *in vitro* results exhibited a high binding affinity for ^99m^Tc-DOTA-(Lys-Dabcyl^6^,Phe^7^)-ARA-290 to the hypoxic cells (*P*<0.001).

***Cardiac ischemia & reperfusion in WISTAR rats***

**Figure 7:**
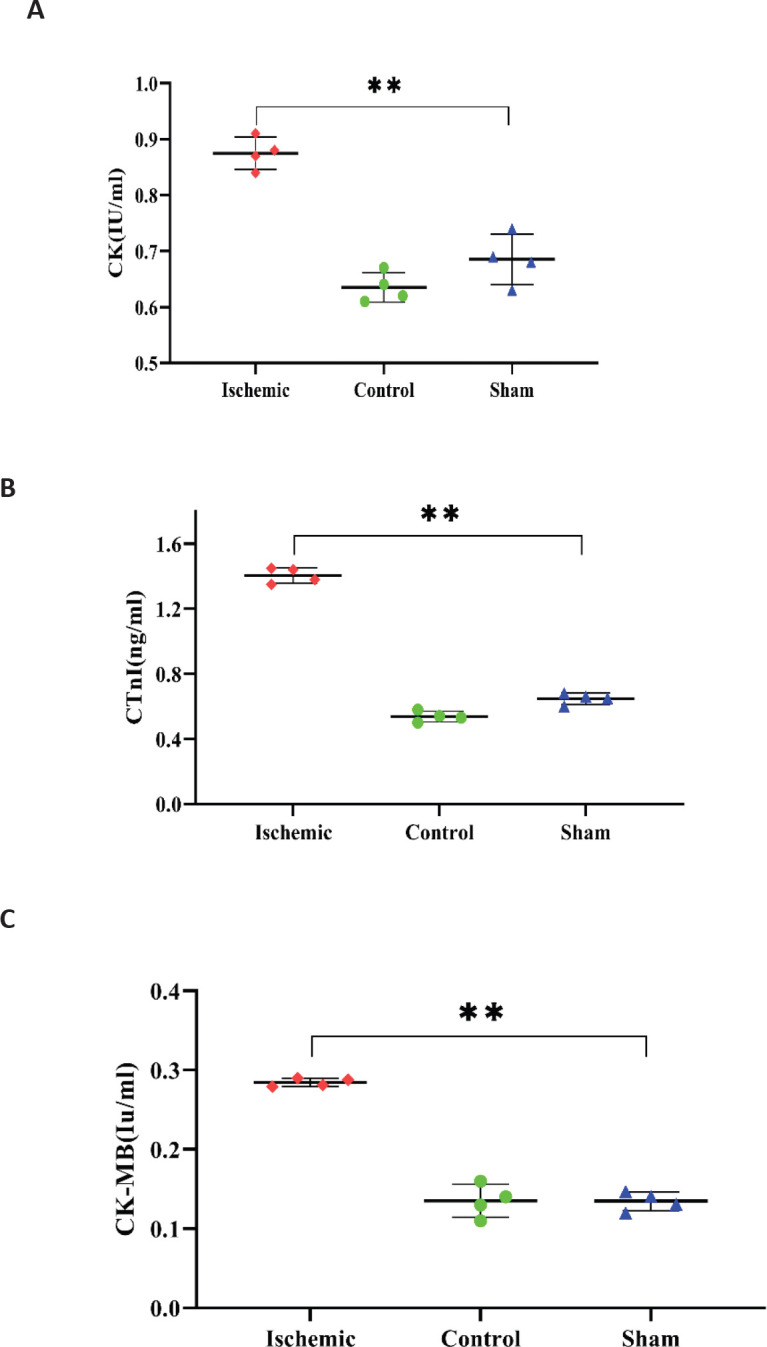
Demonstrative confirm ischemia created by surgery. Cardiac ischemic biomarkers in blood plasma in different groups of rats at 12 hr after LAD ligature: (A) CK, (B) cTnI, (C) CK-MB. Data are means±SD. (n=4, **P<0.01 for ischemic model contrasting to the sham group)

To declare the ischemia created by surgery, cardiac ischemic biomarkers in blood plasma, including CK-MB, cTI, and LDH in different groups of the rats at 12 hr after LAD ligature were measured. Figure 7A shows the normal cardiac cTnI, which is too low in a healthy model; in contrast, it was meaningfully higher in the ischemic rats model (before obstruction of LAD) (*P*<0.01). Besides, each of CK (Figure 7B) and CK-MB (Figure 7 C) in the ischemic group was remarkably high compared with the same and normal group (*P*<0.01). 

***Biodistribution***

Results from biodistribution studies about ^99m^Tc-DOTA-(Lys-Dabcyl^6^,Phe^7^)-ARA-290 in normal rats and the hypoxic model are given in Table 3. In the first 1 hr, in both cases, radiopeptide collecting in the stomach, muscles, lungs, bones, and the salivary glands was very low. Also, through calculation of the % ID/g, we have seen diminished radioactivity over a period of time from half-hour to 6 hr within these organs. It can be inferred from the low activity in this organ that the release of radionuclide from the labeled peptide is low. Accumulation of radioactivity in the cardiac ischemic area was 3.44 ±0.32 ID/g at 0.5 hr post-injection, while simultaneously, this value in healthy cardiac tissue was 0.62 ± 0.15 % ID/g. The heart-to-thyroid radiotracer ratio in the ischemic model was 4.35, 5.29, and 4.68 % ID/g at 0.5, 1, and 3 hr post-injection, respectively. Due to the reperfusion in the ischemic zone after opening the LAD knot, increased oxygen level leads to reduced EPO-BcR receptors on the cell surface, which results in less accumulation of radioactivity in the ischemic area; therefore, activity during 3 hr was decreased from 3.44 ± 0.32% ID at 0.5 hr to 1.36 ± 0.44% ID at 3 hr post-injection. Over time, the accumulation of radioactivity in the kidneys and bladder was increasing (10.58 ± 0.48 and 14.29 ± 0.01%ID at 0.5 and 1 hr post-injection, respectively). This indicates the excretion of radiopeptide from the urinary tract, which confirms the partition coefficient results. The liver uptake was 3.55 ± 0.21 and 4.31 ± 0.52 % ID/g at 1 hr and 3 hr post-injection, respectively, which confirms the radiopeptide metabolism and excretion through the liver. An injection of unlabeled peptides showed a statistically significant decrease in the uptake of radiopeptide in the ischemic region (3.28±0.52 to 0.64% ID/g at 1 hr p.i.)**, **showing EPO-BcR specificity**. **

***Scintigraphy studies***

**Figure 8:**
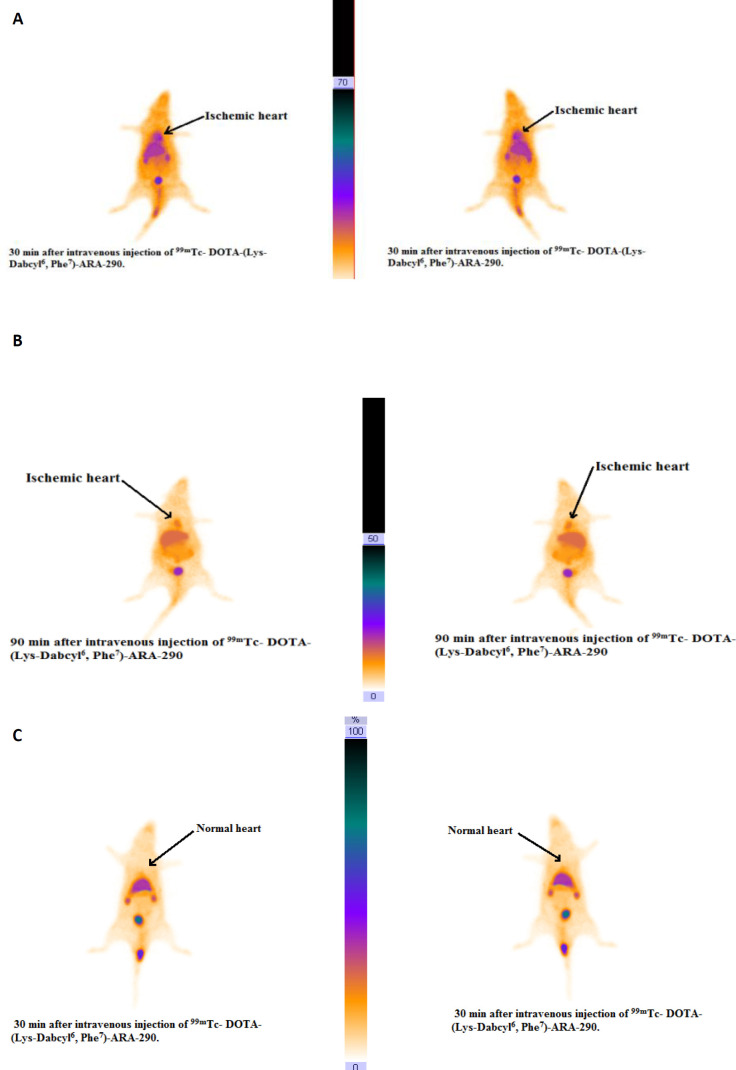
Rat myocardial ischemic scintigraphy images: 30 (A) and 90 (B) min after injecting into the tail vein of ^99m^Tc-DOTA-(Lys-Dabcyl^6^,Phe^7^)-ARA-290. Arrows indicate the high cardiac ischemic accumulation position. (C) Demonstrative normal rat images at 30 min post inection

The ^99m^Tc-DOTA-(Lys-Dabcyl^6^,Phe^7^)-peptide in cardiac ischemic rats exhibited a considerable ischemic region uptake at 30 and 90 min (Figures 8a, b), whereas cardiac accumulation in the normal rat at 30 min post-injection was not detectable (Figure 8C). Figure 8B indicates that during increment of blood flow in the reperfusion phase, ischemic heart uptake gradually reduced. The uptake of high yield of radioactivity in the liver shows metabolism of the peptide, whereas the presence of extraordinary activity in the bladder is indicative of the excretion route of radiopeptide. 

## Discussion

Scientists have given molecular imaging special attention because of diagnosis of diseases and response to the treatment by analyzing changes in biological molecules (30,31). In this regard, different radiotracers depending on the chemical structure and type of emitting radionuclides were synthesized and studied. For the first time in 1990 (4), studies confirmed that in addition to the influence of EPO to control the production and survival of erythrocytes in presence of Epo,  homodimer receptors (Epo-R) also have tissue defending effect by heterodimer EPOR-βcR receptor complex. The EPOR-BcR receptor is immediately induced by hypoxia or tissue inflammation. ARA-290 (pHBSP), an 11 amino acid peptide, taken from the EPO beta subunit, has an anti-hypoxia function by binding to the EPO-BcR receptor. This study aimed to synthesize and investigate DOTA-(Lys-Dabcyl^6^,Phe^7^)-ARA-290s labeled by technetium-99m to address cardiac ischemia. Fleming *et al*. (32), developed nitroimidazole analogs that were labeled with F18, including [^18^F] fluoroetanidazole (FETA) and [^18^F] fluoromisonidazole (FMISO) as a PET imaging of intratumoral hypoxia which is routinely used in nuclear medicine centers. Other PET radionuclides attached to the imidazole derivatives were investigated successfully in hypoxic imaging (33). The use of imidazole derivatives labeled with ^18^F isotope for tissue hypoxia imaging has two constraints: delayed clearance from normal cells (poor image contrast) and less radioactivity accumulation in hypoxic tissues (34). Opposite to these compounds, the use of small peptide (ARA-290) as a technetium-99m pertechnetate-based substance imaging agent has a number of different benefits, such as very low acute toxicity, biodegradability, easy production, and ability to attach cyclic chelator (BFCA) (35). 

Myocardial SPECT imaging as a nuclear cardiological scan has been used in many procedures for ischemic heart diseases (36). In the mid-1970s, THALLIUM-201 (201TI) imaging has been used to ascertain regional myocardial perfusion models at rest, stress, and acute myocardial infarction, but because of the concerns about medical exposure to ionizing radiation and lower image quality, ^201^Tl was substituted by ^99m^Tc-labeled perfusion agents (^99m^Tc-sestamibi and ^99m^Tc-tetrofosmin). However, these SPECT agents show poor pharmacokinetic features with reducing uptake with higher flows (37, 38). Among the advantages of ^99m^Tc-DOTA-ARA-290 over classic cardio radio-pharmaceutics are independence on blood perfusion, biodegradability, and peptide’s extraordinary properties such as high flexibility in chemical changes, wide variety of biological activity and pleasant pharmacokinetics, including high specific peptide-receptor binding, and quick exertion from blood and non-target organs. Thus, after synthetic modifications and molecular engineering improve the half-life or coupling to the radionuclide, peptides are widely used as radiopharmaceuticals for diagnosis and treatment (39). There are various methods of inhibiting proteolysis of peptides: using modified amino acids, thioesters cyclization, C-terminal amidation or N-terminal acylation, and using peptide mimetic groups (12, 13). During synthesis of the peptide to increase the stability of ARA-290, Fmoc-Lys(Dabcyl)-OH and Fmoc-Phe-OH were replaced in lieu of Fmoc-Arg^6^ and Fmoc-Ala^7^ sequentially, then conjugated to DOTA-NHS-ester. DOTA-NHS-ester can be chelated with various types of radionuclides and easily reacts with carboxyl groups in peptides, proteins, and antibodies (17). In this framework, Yang *et al*. (40, 41), manipulated the thioether-cyclized approach to enhance the metabolic stability of ARA-290. They were able to increase *in vivo* stability from 2 to 45 min. In another study conducted by Dumont *et al*. (13), after conjugation of ARA-290 with PEG, desirable stability was obtained.

To achieve high radiochemical purity, several changes to the ideal mixture of different ingredients, including peptide, SnCl_2_, and pH were regularly tested. The highest purity was obtained at pH similar to body pH (7.5). In acidic pH, the radiochemical purity was probably reduced because pairs of discontinuous electrons of N, O atoms contained in the chelator are covered with proton and prevent binding to ^99m^TcO^- ^_4_ (42). At the optimal pH and SnCl_2_ levels, increasing the amount of DOTA-(Lys-Dabcyl^6^,Phe^7^)-ARA-290 from 5 to 50 μg RCP % was improved. The specific activities decreased from 385.02 to 38.01 MBq, respectively. Unlike ^99m^Tc as SPECT radioisotope, producing PET radionuclides needs cyclotrons machines which are expensive and are difficult to ship, and also due to short half-life, some of them are ineffective for acquiring delayed images (43, 44). So, we used ^99m^Tc to label the synthesized DOTA-peptide.

*In vitro* hypoxic cellular binding of ^99m^Tc-DOTA-(Lys-Dabcyl^6^,Phe^7^)-ARA-290 revealed the very noticeable ability of the radiopeptide tendency to EPO-BcR receptor-positive cells (*P*<0.01). In this study, by culturing the cells in a normoxic situation and blocking experiments on H9c2 hypoxia cells, radiopeptide’s specific affinity to EPOR-βcR was approved. 

Results from biodistribution analyses showed the highest observed uptake in kidneys (14.29±0.01 ID/g at 1 hr post-injection), and this proves that the excretion pathway of the radiopeptide is into kidneys, which is in agreement with the result of the substance Log p. Based on the evidence in Table 3, the activity uptake of liver tissue is insignificantly high (3.55% ID/g at 0.5 hr), also higher radioactivity was detected in the SPECT scans for two reasons; first, overlapping organs and second, hepatic metabolism of peptides and amino acids in the liver (45). Radioactivity in the stomach and thyroid was negligible which is in conformity with *in vitro* stability and low ^99m^Tc-free in *in vivo* results. In a healthy heart, ^99m^Tc-DOTA-(Lys-Dabcyl^6^,Phe^7^)-ARA-290 complex was 0.31±0.18 %ID/g at 1 hr which displays a lack of absorption of radiotracer by normal heart. Blocking tests revealed that ischemic cardiac uptake of radioligands is EPO-βcR mediated, whereas radiotracer uptake in other organs such as the liver and kidney is not EPO-βcR mediated. The images obtained from SPECT in the early time after injection of the radiotracer showed that the ischemic cardiac region was early detectable. In our recently published paper (46), ^99m^Tc- dendrimer-G_2_-pHBSP peptide was used for targeting ischemic heart disease but compared with the previous study, in this work, we used the appropriate DOTA-chelator instead of large muscular weight and non-specific chelator PEGylated dendrimer-G_2_. On the other hand, we synthesized Fmoc-Lys(Dabcyl)-OH as an amino acid derivative. Therefore, in the present study, we could improve the radiochemical purity of 94% to 96% by using a DOTA chelator and eliminate the cost of buying the Fmoc-Lys (Dabcyl)-OH derivative of the amino acid.

Several investigations of various diseases, including multiple infarcts cerebral ischemia, critical limb ischemia, cardiac ischemic model in mice, and ischemia-reperfusion injury of kidney in rats are in accord with the results of our *in vitro* studies and show pHBSP to be effective and applicable (47-49). Furthermore, these results confirm that ^99m^Tc-DOTA-(Lys-Dabcyl^6^,Phe^7^)-ARA-290 may be a new molecular imaging agent for early detection of ischemic heart disease.

**Table 1:** Analytical data DOTA-(Lys-Dabcyl^6^,Phe^7^)-ARA-290

Compound				
ARA-290	Mass spectrum		RP-HPLC	
	Designed mass (g)	Detected mass (g)	RT (min)^a^	Purity (%)
1,959.33		1,960.25 [M +H]^+^	17.36	>97

^a^ RT retention time

**Table 2:** Effect of DOTA-(Lys-Dabcyl^6^,Phe^7^)-ARA-290 ligand on radiolabeling efficacy in the presence of 925 MBq ^99m^TcO4− (mean ± SD, n=3)

DOTA-(Lys-Dabcyl^6^,Phe^7^) -ARA-290 (µg)	**% RCP**	**Specific activity (MBq/nmol)**
**5**	82.04	385.02
**10**	88.25	190.32
**15**	91.89	126.88
**20**	96.02	94.96
**25**	96.24	75.22
**30**	96.48	63.39
**40**	96.65	47.53
**50**	96.89	38.01

(n=3,*P<0.05, **P<0.01, ***P<0.001 for hypoxic condition comparison in normoxic condition)

**Table 3:** Biodistribution of 99mTc-DOTA-(Lys-Dabcyl6,Phe7)-ARA-290 in rats (% injected dose per gram organ ± SD, n= 3)

360 min	180 min	60 min blc		60 min	30 min	Organ
0.12± 0.87	0.44± 0.28	1.02 ± 0.58	0.96 ± 0.16		1.52 ± 0.29	Blood
0.14 ± 0.23	0.21 ± 0.32	0.34 ± 0.31	0.31 ± 0.18		0.62 ± 0.15	Heart
0.32 ± 0.22	0.52 ± 0.19	0.75 ± 0.02	0.76 ± 0.77		0.94 ± 0.58	Lung
2.08 ± 0.28	3.49 ± 0.09	4.31 ± 0.52	4.28 ± 0.15		3.55 ± 0.21	Liver
0.44 ± 0.29	1.16 ± 0.22	2.29 ± 0.52	2.31 ± 0.12		2.56 ± 0.21	Spleen
0.32 ± 0.19	0.54 ± 0.28	0.74 ± 0.36	0.71 ± 0.31		0.88 ± 0.44	Stomach
2.35 ± 0.25	2.89 ± 0.08	3.21 ± 0.02	3.22 ± 0.78		1.73 ± 0.10	Intestines
4.06 ± 0.43	8.58 ± 0.10	14.58 ± 0.11	14.29 ± 0.01		10.58 ± 0.48	Kidneys
0.17 ± 0.31	0.25 ± 0.87	0.42 ± 0.51	0.42 ± 0.84		0.53 ± 0.42	Muscle
0.18 ± 0.81	0.29 ± 0.29	0.61 ± 0.21	0.62 ± 0.15		0.79 ± 0.41	S.g & Th^a^
0.16 ± 0.29	0.32 ± 0.21	0.51 ± 0.44	0.53 ± 0.21		0.75 ± 0.41	Bone
1.08± 0.94	1.36 ±0.44	0.64 ± 0.06	3.28± 0.52		3.44± 0.32	Isch heart^b^
6.01	4.68	1.04	5.29		4.35	Isch heart/Th
3.37	2.61	0.85	4.31		3.65	Isch heart/Lung

^a^ Salivary gland and thyroid are abbreviated as S.g and Th; b Ischemic heart and; c bl block

## Conclusion

According to the results of analytical HPLC and mass spectroscopy, DOTA-(Lys-Dabcyl^6^,Phe^7^)-ARA-290 synthesis by the solid-phase method has high purity. Adjustment of labeling ingredient showed remarkable radiochemical purity and stability even up to 6 hr post labeling. ^99m^Tc-DOTA-(Lys-Dabcyl^6^,Phe^7^)-ARA-290 showed a statistically significant affinity to binding the EPO-BcR receptor on the hypoxic cell surface. Synthesized radiopeptides showed high accumulation in the ischemic cardiac region as a positive EPO-BcR receptor followed by excretion by the kidney. This research concludes that the ^99m^Tc-DOTA-(Lys-Dabcyl^6^,Phe^7^)-ARA-290 may provide possible use as a suitable radiolabeled peptide in the detection of SPECT cardiac ischemic imaging.

## Authors' Contributions

NM and MH Study conception and design; MA Data processing, collection, performing experiments and study conception and design; ABR Critical revision and editing of the article; NGH Analysis and interpretation of results, Supervision, funding acquisition, analysis and interpretation of results and study conception and design.

## Conflicts of Interest

The authors declare that there are no conflicts of interest.
